# The role of tau protein in HIV-associated neurocognitive disorders

**DOI:** 10.1186/1750-1326-9-40

**Published:** 2014-10-10

**Authors:** Lecia AM Brown, James Scarola, Adam J Smith, Paul R Sanberg, Jun Tan, Brian Giunta

**Affiliations:** Department of Molecular Pharmacology and Physiology, Morsani College of Medicine, University of South Florida, Tampa, FL 33613 USA; Department of Psychiatry and Behavioral Neurosciences, Neuroimmunology Laboratory, Morsani College of Medicine, University of South Florida, 3515 E. Fletcher Ave., MDT14, Tampa, FL 33613 USA; Center of Excellence for Aging and Brain Repair, Department of Neurosurgery and Brain Repair, Morsani College of Medicine, University of South Florida, Tampa, FL 33613 USA; Department of Psychiatry and Neurosciences, Rashid Developmental Neurobiology Laboratory, Silver Child Development Center, Morsani College of Medicine, University of South Florida, Tampa, FL 33613 USA; James A. Haley Veterans Administration Hospital, Tampa, FL 33612 USA

**Keywords:** HIV, Cognitive, Tau, Neurodegeneration, Inflammation, cART

## Abstract

Given the increased life expectancy of human immunodeficiency virus (HIV) infected individuals treated with combination antiretroviral therapy (cART) and the ongoing inflammation observed in the brains of these patients, it is likely that premature neurodegeneration as measured by phospho-tau (p-tau) or increased total tau (t-tau) protein may become an increasing problem. This review examines the seven human studies that have occurred over the past 14 years measuring p-tau and/or t-tau in cerebrospinal fluid (CSF) or *via post-mortem* brain immunohistochemistry. Although not all studies are in agreement as to the changes in p-and t-tau in HIV infected patients, HIV persists in the brain despite cART. Thus is it is suggested that those maintained on long-term cART may develop tau pathology beyond the extent seen in the studies reviewed herein and overtime may then reach the threshold for clinical manifestation.

## Introduction

### HIV-associated neurocognitive disorders (HAND)

More than 33 million people worldwide are infected with the human immunodeficiency virus (HIV) [1]. HIV-1 infection of the central nervous system (CNS) can result in cognitive, motor, and behavioral deficits, collectively termed HAND [2, 3]. These deficits present along a spectrum from asymptomatic neurocognitive impairment (ANI) to HIV associated dementia (HAD). The latter is also termed HIV associated dementia complex (HADC), AIDS dementia complex (ADC), or HIV-encephalitis (HIVE; a diagnosis which can be made only upon brain autopsy exam) depending on the research report [3]. Soon after infection by the HIV, it rapidly moves into the brain *via* infected monocytes and lymphocytes [4] and, despite combination antiretroviral therapy (cART), persists in parenchymal microglia as well as the perivascular macrophages [5–7]. As HIV is unable to productively infect neurons, neuronal cell damage is largely promoted by neurotoxins secreted by these infected and/or activated macrophages, microglia, and astrocytes [5]. In spite of the fact that the clinical severity of HAND has been significantly reduced due to the widespread utilization of cART, the prevalence and associated morbidity still remains high (~50% [8, 9]). Why HAND still persists in the current era of cART, even in patients effectively controlled for systemic viremic load, is incompletely understood. Recent evidence suggests prolonged inflammation in both the brain and periphery may be responsible [10–13].

Chronic HIV infection promotes neuroinflammation and leads to neuronal death and damage [14]. In addition, it has been reported that subjects treated with combination cART have high levels of neuroinflammation, especially in the hippocampus [13, 15]. This type of neuroinflammation in the form of microglial activation is associated with a number of neurodegenerative diseases including Alzheimer’s disease (AD), Pick’s disease, and hereditary frontotemporal dementia with Parkinsonism linked to chromosome 17 (FTDP-17) [16, 17].

Since the introduction of cART the neuropathology associated with HIV has changed. The prevalence of some opportunistic conditions including *Cytomegalovirus* and toxoplasmosis has decreased while that of others such as progressive multifocal leucoencephalopathy and lymphoma appears to have not changed [15, 18, 19]. The effect of cART on neurocognitive decline and dementia is, on the other hand, less clear, despite a reduced prevalence of the most severe form of HAND; HAD. While some studies report improved cognitive function in cART-treated individuals [20–24], others suggest cognitive impairment is still a significant clinical problem [9, 24–32].

Regardless of the effects of cART directly on HAND, HIV patients who are given this therapy are now living much longer. As humans age, HIV infected or not, there is an increase in the prevalence of signs of neurodegeneration [33] even in otherwise healthy individuals [34]. Since 2000 there has been an array of reports suggesting the possibility of advanced brain aging in the form of AD-like pathology in HIV patients [35–40]. Most of the recent literature indicates that there is an increased risked of advanced brain aging but perhaps not in the exact pattern of true AD. Rather the reports signal an increase in certain, but not all forms of the key proteins of AD, amyloid-beta (Aβ) and tau. This review focuses on tau protein and begins to identify what influence both chronic HIV infection and long-term cART will have on the process of advanced brain aging in the form of abnormal tau pathology and associated functional deficits.

### Tau protein

Central to the formation and subsequent stabilization of microtubules as well as the movement of organelles along axons and dendrites, tau is a microtubule-associated protein which is largely expressed in central nervous system (CNS) neurons [41]. Inflammatory stimuli can facilitate tau phosphorylation [42–44] although it remains unclear where phosph-tau is the cause, result, or merely an correlation with inflammation. The hyperphosphorylation of tau has been associated with neurodegeneration [45–47]. Further, hyperphosphorylated tau (p-tau) can undergo cellular accumulation leading to the formation of insoluble neurofibrillary tangles (NFTs) and neuropil threads [48] and thus in some transgenic models of AD, deletion of tau is protective [49, 50].

In very young healthy individuals hyperphosphorylated tau is not commonly detected [51]. It has been reported that by the age of 55, half of individuals show some evidence of tau in the entorhinal cortex (EC) [51]. In these early stages, hyperphosphorylated tau is usually only found in the EC in non-demented individuals. By the age 75, almost all brains contain some hyperphosphorylated tau which has spread to involve the hippocampus (CA1) and neocortex. It should be noted however that tau accumulation is not an inevitable consequence of ageing since studies have identified extremely old, non-demented individuals with only mild tau pathology at autopsy [52]. Although a full understanding of any pathophysiological importance of increased tau (t-tau) is not yet established, it is seen in AD and to a lesser extent in vascular dementia and other neurodegenerative disorders [53]. Therefore, given the increased life expectancy of HIV-infected individuals treated with cART and, age being a major risk factor for increased phospho-tau, it likely that premature brain ageing as measured by p-tau or increased t-tau protein may become an increasing problem.

### Epidemiology of Tau protein in HIV infection

**Figure 1:**
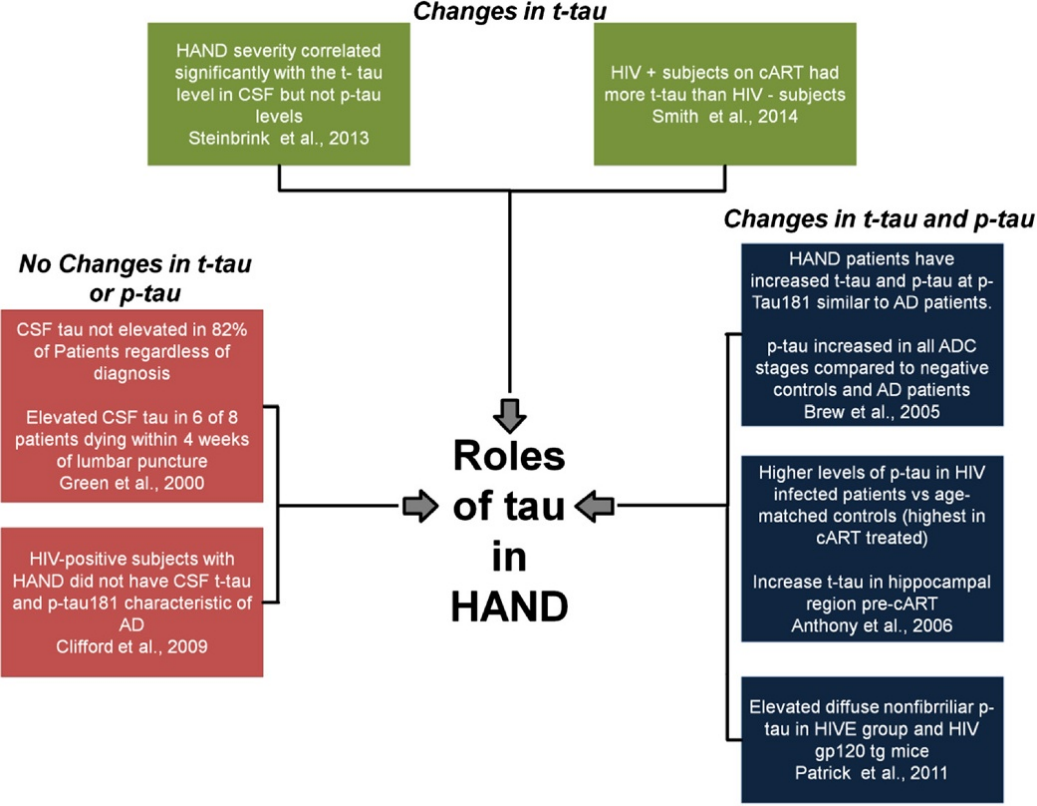
**Synopsis of clinical studies examining the relationship between tau and HIV infection and/or HAND.**

Over the past 14 years there have been several epidemiological studies examining cerebrospinal fluid (CSF) and *post- mortem* brain samples for changes of p-tau or t-tau in HIV infected patients (Table 1, Figure 1). Some studies have taken cART into account [39, 54–57] while others have not [35, 37]. A study by Green and colleagues prospectively measured CSF t-tau in 76 consecutive HIV infected patients who had acute neurological episodes. Twenty four patients had HADC, 10 had lymphoma, 20 had cerebral infections; 22 patients had miscellaneous conditions, including nine with self-limiting headache/fever [35]. The subjects had a median age of 37 years. Past history of cART was not taken into account. CSF tau levels were acquired by enzyme linked immunosorbent assay (ELISA) (INNOTEST, Fujirebio Europe/Innogenetics, Ghent, Belgium) [58] with an upper limit of normal in CSF set at 315 pg/ml and a lower limit of detection set at 75 pg/ml (Table 1). Results indicated CSF tau was not elevated in the majority (62/76) (82%) of patients, regardless of their CD4 lymphocyte count or their clinical diagnoses. Elevated CSF t-tau was significantly associated with poor outcome as six of eight patients who died within 4 weeks of lumbar puncture had elevated t-tau [35].

**Table 1:** **Epidemiological studies of p-tau and t-tau in HAND**

Author, Year	Study	Subjects	Technique used	Tau detection range (pg/ml)	p-tau and/or t-tau result
Green et al. [35]	Cerebrospinal fluid tau concentrations in HIV infected patients with suspected neurological Disease	1) Mean age: 37 yr. with acute neurological episodes	ELISA (INNOTEST, Fujirebio Europe/Innogenetics, Ghent, Belgium)	t-tau: 75-316	1) CSF tau not elevated in 82% of patients, regardless of clinical diagnoses.
		2) N = 76 (24-HAD, 10 lymphoma, 20 cerebral infarctions, 22 miscellaneous conditions such as headache)			2) Elevated CSF tau was associated with poor outcome as 6 of 8 patients who died within 4 weeks of lumbar puncture.
		3) cART not accounted for			
Brew et al. [37]	CSF amyloid beta42 and tau levels correlate with AIDS dementia complex	1) Mean age: 43 years	ELISA (INNOTEST, Fujirebio Europe/Innogenetics, Ghent, Belgium)	Upper limits not reported /lower limits: t-tau: 75, p-tau:16	1) HAND subjects had significantly increased t-tau and p-tau at residue 181 (p-Tau181) at concentrations similar to patients with AD
		2) N = 101 HIV positive subjects with or without ADC with 20 moderate to severe AD subjects as positive control			2) p-tau levels significantly increased in all of the ADC stages compared with the negative controls and the AD patients.
		3) cART not accounted for.			
Anthony et al. [54]	Accelerated Tau deposition in the brains of individuals infected with human immunodeficiency virus-1 before and after the advent of highly active anti-retroviral therapy	1) Mean age: 40 for HIV- control group and HIV positive cases, 70 for HIV negative control group and 40 for HIV negative control group B	Immunohistocytochemistry (TSA was used in instances where avidin-biotin complex ABC was not sensitive enough, with DBA for visualization.)	NA: AT8 antibody used on paraffin sections	1) Higher levels of p-tau in HIV infectedsubjects vs. aged matched controls.
		2) N = 34			2) Greatest levels of p-tau were noted in cART-treated subjects.
		3) Nine cART treated subjects with excellent compliance for at least 18 months with pre-symptomatic HIV or AIDS and 20 pre-cART HIV subjects			3) Increased t- tau in hippocampal region of pre-cART HIV-infected groups compared to HIV-negative age-matched controls
Clifford et al. [39]	CSF biomarkers of Alzheimer disease in HIV-associated neurologic disease	1) Mean age: normal cognition control group (50 years), HIV + normal cognition (43 years), HAND subjects (48 years), mild AD subjects (74 years)	ELISA, (INNOTEST, Fujirebio Europe/Innogenetics, Ghent, Belgium)	Not reported	HIV-positive subjects with HAND did not have CSF t-tau and p-tau181 characteristic of AD
		2) N = 188 (50 control, 68 AD subjects, 21 Neuro-normal HIV positive subjects and 49 HAND subjects.			
		3) HIV patients were not treated with cART.			
Patrick et al. [55]	Increased CDK5 expression in HIV encephalitis contributes to neurodegeneration via tau phosphorylation and is reversed with Roscovitine	1) Mean age: HIVE patients - 43.13 years, Non HIVE subjects (48.38 years )	Immunohistochemistry	N/A:AT8 and PHF-antibody detection on *post-mortem* brain	Elevated diffuse nonfibrriliar p-tau in HIVE group and HIV gp120 tg mice.
		2) N = 16: 8 HIVE subjects, 8 HIV positive without HIVE	(TSA was used in instances where avidin-biotin complex ABC was not sensitive enough, with DBA for visualization.)		
		3) cART not accounted for			
Steinbrink et. al. [56]	Cognitive impairment in HIV infection is associated with MRI and CSF pattern of neurodegeneration	1) Mean age: 45 ± 10 years	ELISA (INNOTEST, Fujirebio Europe/Innogenetics, Ghent, Belgium)	Not reported	1) Significant correlation between HAND and t-tau but not p-tau
		2) N = 94. All patients were HIV positive with varying levels of neuropsychological performance,			2) HAND severity correlated significantly with the t- tau level in CSF but not p-tau levels
		3) 68% cART treated			
Smith et al. [57]	Brain viral burden, neuroinflammation and neurodegeneration in HAART-treated HIV positive injecting drug users	1) Mean age: 45.8 years for HIV+, 42.2 years for HIV-	Immunohistochemistry	NA: AT8 on *post-mortem* brain	1) IDU had more t- tau vs. non-DU
		2.) N = 20 :10 HIV + (6 IDU, 4 non-IDU),10 HIV (6 IDU, 4 non-IDU)	(TSA was used in instances where avidin-biotin complex ABC was not sensitive enough, with DBA for visualization.)		2) HIV + subjects had more t-tau than HIV -, but these differences did not achieve statistical significance.
		3) HIV + patients treated with cART for up to 7.9 years

Subsequently there was an analysis of the prevalence of CSF t-tau and p-tau concentrations from 101 HIV-1 seropositive individuals, some of whom had ADC [37]. The CSF from 20 patients with moderate to severe AD was used as a positive control [37]. CSF from 20 patients not known to have HIV or AD was also analyzed. CSF t-tau was quantified using an hTau antigen assay, and p-tau concentrations were determined using a Phospho-Tau181 (p-tau181) assay that recognizes the p-Tau at Threonine-181. Both were measured using the Innotest ELISA with lower limits of 75 and 16 pg/mL. They found HAND subjects had significantly increased t-tau and p-tau at concentrations similar to patients with AD, suggesting that ADC may be associated with an AD-like process [37]. Overall, CSF t-tau levels were significantly increased in ADC Stage 2 while CSF p-tau levels were significantly increased in each of the ADC stages compared with the negative controls and the AD patients. The authors noted that these data indicate patients with ADC have significantly increased t-tau and p-tau concentrations which are of the same magnitude as found in AD patients [37]. In eight ADC patients (mean age 43 years), CSF t-tau was increased [37]. This is in contrast to the previous study by Green and colleagues [35] where 24 ADC patients (median age 37 years) were analyzed, finding only 5 with increased levels. One explanation for these conflicting results relates to the age of the patients [35] and this confounder also occurs in other studies in this review. In general studies indicating CSF t-tau concentrations that were increased were composed of older individuals (mean age of 43 years or older). Conversely those with negative results had a younger mean age, in this case [37] approximately 37 years. The study by Green and colleagues [35], did show a non-significant trend to higher values in those patients 40 years of age and older. Furthermore, Green and colleagues study [35] likely included more subjects with more severe ADC because which is in agreement with the results of Brew and colleagues who showed a relationship to Stage 2 ADC [37] (Table 1).

Following, there was an investigation into investigated the prevalence of tau-associated neurodegeneration in nine cART-treated individuals who had a history of excellent therapeutic compliance for at least 18 months and compared 20 pre-cART HIV-infected subjects and 14 control cases [54]. The pre-cART groups included: pre-symptomatic HIV-positive and AIDS cases. The nine cART-treated cases had a history of excellent therapeutic compliance for at least 18 months. Two groups of HIV-negative control cases with no CNS pathology were selected to provide a baseline. The first group was age matched (mean age 40) with the HIV cases while the second group represented older individuals (mean age 70) who had no history of cognitive decline or other neurodegenerative disorder. This older group allowed for analyzing the extent of any premature ageing effect in a much younger HIV cohort. Paraffin blocks of hippocampus and pons were selected for each case. Immunohistochemistry was performed using AT8 antibody, which recognizes p-tau at serine 202. Tyramide signal amplification (TSA) was used to enhance staining in experiments because the standard avidin-biotin complex (ABC) techniques were not sensitive enough for several of the antibodies. Diaminobenzidine (DAB) was then used to visualize all antibodies. Results of this study showed higher levels of p-tau in the EC, hippocampus, and neocortex in HIV infected subjects compared to aged matched controls. Interestingly, the greatest levels of p-tau were noted in cART-treated subjects. Increased levels of t-tau were also seen in the hippocampal region of pre-cART HIV-infected groups compared to HIV-negative age-matched controls [54]. The increases in tau were independent of a history of drug abuse, although the number of cases studied in each group was low when subdivided into drug abusers and non-drug abusers. This is interesting given past finding of increased levels of p-tau in young HIV-negative drug abusers (mean age 27) [59]. The group of pre-symptomatic subjects studied were all HIV-positive drug abusers and therefore in this group it was not possible to determine whether p-tau levels were increased compared to controls as a result of drug abuse, as reported by Ramage et al., [59] or whether factors related to HIV infection were responsible. No evidence of significant head injury in patients’ life history or on routine neuropathological examination was found and it was thus conclude that head injury was unlikely to be a contributing factor in the observed tau pathology [54]. The authors note that if two control groups represent different ends of the spectrum of normal deposition of hyperphosphorylated tau in non-demented individuals, then these results suggest that HIV-infected individuals have shifted significantly away from normal age-matched controls towards the levels seen normally in ‘old age’ [54]. This would suggest these individuals are subject to advanced brain aging, which is of particular concern in the cART-treated group and may promote future HAND [33]. Thus those maintained on long term cART may develop tau pathology beyond the extent seen in this study and that this may then reach the threshold for clinical manifestation [54].

Clifford and colleagues [39] examined CSF from a total of 188 subjects clinically categorized with normal cognition from the general population (mean age 50 years), HIV + subjects with normal cognition (mean age 43 years), HIV + subjects with impaired cognition (mean age 48), or presumed HIV - subjects with mild AD (mean age 74). CSF samples were analyzed for t-tau, and p-tau by ELISA (INNOTEST, Fujirebio Europe/Innogenetics, Ghent, Belgium), however, upper and lower limits of detection were not supplied in the report. In contrast to Brew and colleagues [37], HIV-positive subjects with HAND did not have CSF t-tau and p-tau characteristic of AD however, this report in congruence with Brew and colleagues analyses in which also indicates t-tau was significantly elevated in CSF of this HIV positive population versus non-HIV infected subjects [37]. Overall there was a trend for HAND patients to have higher p-tau and t-tau than HIV positive non-HAND patients. It is notable that this report of non-significantly elevated p-tau in the AD patients is also not in agreement with the AD literature.

Using both an epidemiological approach to identify tau pathology in HIV infection, and then modeling this phenomenon *in vivo* can lead to useful prevalence and mechanistic data regarding the influence of abnormal tau on HAND. Patrick and colleagues [55], using double labeled using the Tyramide Signal Amplification-Direct (Red) system (NEN Life Sciences, Boston, MA). Using this system, FITC tagged p-tau immunohistochemistry antibodies: AT8 and PHF-1 were analyzed, in the brains of 43 patients with HIVE (median age 43.13 years) and in gp120 Tg mice, showing that increased expression of cyclin-dependent kinase 5 (CDK5) can lead to neurotoxicity *via* promotion of abnormal tau phosphorylation. Researchers utilized FITC tagged manufactured p-tau immunohistochemistry antibodies: AT8 (Ser 202) and PHF-1, which detects p-tau at Ser396 and Ser404. Subjects were excluded if they had a history of CNS opportunistic infections or non-HIV-related developmental, neurological, psychiatric, or metabolic conditions that might affect CNS functioning. For inclusion, a total of 16 age-matched cases were identified with and without encephalitis or other complications. cART status was not taken into account. Overall the resulting data are consistent with other studies indicating aberrant activation of the CDK5 [60, 61] and glycogen sythanse kinase (GSK)3β [60, 62–65] signaling promoting neurodegenerative changes in HAND patients. The p25/CDK5 complex associates with NFT in AD patients [66], and CDK5 has been shown to abnormally phosphorylate tau [67]. These are recognized by the AT8 and PHF-1 antibodies [68]. Both of these phosphorylation epitope-specific tau antibodies were elevated in patients with HIVE and in gp120 Tg mice. The patterns of AT8 and PHF-1 immunostaining in HIVE patients differed from what has been reported in AD patients. That is, in advanced AD cases the p-tau immunoreactivity is associated with dystrophic neurites, neuropil threads, and NFT [69–73]. On the other hand in the HIVE cases and in gp120 Tg mouse model there was diffuse nonfibrillar p-tau immunostaining detected in neurons and throughout the neuropil. Increased CDK5 and p35/p25 immunoreactivities were also detected throughout the neuropil of HIVE brains and gp120 Tg mice, indicating the expression of these proteins in cellular compartments that extend into the dendritic arbor and synapses. Data on whether the patients were on cART was not provided. The diffuse pattern of p-tau immunostaining was similar to what has been described in patients with preclinical AD or in the pretangle stage [70, 74], suggesting that in HIVE some of the initial triggering events are present [55] which is in agreement with the previous study by Anthony et al. [54] and suggests HIV patients, whether on cART or not, may develop tau pathology beyond the extent seen at the age relatively young ages of the patients of these studies and that tau pathology may reach the threshold for clinical manifestation as the aging process continues [54].

A recent cross-sectional investigation examined 94 patients (mean age 45 ± 10 years) *via* CSF analysis for t-tau and p-tau [56]. This study only enrolled patients who never received cART because of unimpaired immune state or who were taking their first cART regimen. In total, 68% of the cohort was cART-treated. Seventy-two percent (72%) of the patients with cART were virologically suppressed. Upper and lower limits of detection were not supplied for the t-tau or p-tau ELISA assays (INNOTEST, Fujirebio Europe/Innogenetics, Ghent, Belgium). Patients with signs of opportunistic CNS infection by MRI or CSF analysis were excluded from further analysis as well as those with psychiatric disorders or severe impairment of consciousness. A significant correlation between HAND and t-tau was uncovered, but not for p-tau [56]. Interestingly HAND severity as measured by the Memorial Sloan-Kettering scale, HIV dementia scale and Mosaic test correlated significantly with the total tau level in CSF but not p-tau levels [56]. Although measures of global brain atrophy via MRI in this study did not significantly correlate with the increase in t-tau, the results suggest t-tau might be a non-specific marker of ongoing subcortical CNS damage particularly in the region of the periventricular white matter and the basal ganglia in HIV infection.

A most recent study by Smith et al. [57] examined p-tau in ten HIV positive and ten HIV negative subjects. A portion of both groups were IDU. All HIV positive patients had been treated with cART for up to 7.9 years and most had received Zidovudine before the start of cART. Cases with opportunistic infections or tumors in the brain were excluded from this study. All six HIV positive IDU, but only one HIV positive non-DU, were HCV positive while the HIV negative IDU and non-IDU were all HCV-negative. Using the AT8 (Ser202) antibody as the primary method for tau quantification, the results revealed declining positivity for tau (neuropil threads and NFT) across all four groups, with IDU having more tau than the non-DU and HIV positive subjects more than HIV negative, but these differences did not achieve statistical significance. A likely reason for the non-significance may be that the study was underpowered [57].

## Conclusion

Together, this review suggests that the measurement of t-tau and p-tau in CSF maybe a useful tool for monitor HAND risk at relatively young ages, or for predicting prognosis in later ages (Table 1). Though differing methods of tau quantification were utilized across a portion of the reviewed reports, the research is beneficial in emphasizing the relationship between the presence of t-tau and p-tau in the brain or CSF and the patient’s HAND status. Because of the aging of the HIV-positive population, this biomarker may well be of increasing utility. Although the incidence of HAD has decreased with the widespread use of cART, patients continue to experience varying degrees of HAND [75]. Since even mild impairment affects quality of life [76], the neurological consequences of HIV infection have gained increased attention, as has the need to distinguish those at risk for impairing neurodegeneration through dynamic changes in tau. Future studies with larger numbers of patients across a wider age range may fine-tune this suggested form of evaluation for HAND risk and as a possible treatment target.

## Abbreviations

Aβ: Amyloid-beta
AD: Alzheimer’s disease
ADC: AIDS dementia complex
AIDS: Acquired immunodeficiency virus
ADC: AIDS dementia complex
ANI: Asymptomatic neurocognitive impairment
CNS: Central nervous system
cART: Combination antiretroviral therapy
CSF: Cerebrospinal fluid
CDK5: Cyclin-dependent kinase 5
EC: Entorhinal cortex
GSK: Glycogen synthase kinase
HAD: HIV associated dementia
HADC: HIV associated dementia complex
HAND: HIV associated neurocognitive disorders
HIV: Human immunodeficiency virus
HIVE: HIV-encephalitis
IDU: Intravenous drug user
NFT: Neurofibrillary tangle
p-tau: Phospho tau
t-tau: Total tau.
